# A Study of Polycrystalline Silicon Damage Features Based on Nanosecond Pulse Laser Irradiation with Different Wavelength Effects

**DOI:** 10.3390/ma10030260

**Published:** 2017-03-03

**Authors:** Jiangmin Xu, Chao Chen, Tengfei Zhang, Zhenchun Han

**Affiliations:** 1 School of Mechanical Engineering, Jiangsu University of Science and Technology, Zhenjiang 212013, Jiangsu, China; snowden_chen@163.com (C.C.); tengfeizhang123@126.com (T.Z.); 2 School of Mechanical Engineering, Jiangsu University, Zhenjiang 212013, Jiangsu, China; 13775327118@163.com

**Keywords:** laser wavelength, polysilicon, laser damage, thermal shock

## Abstract

Based on PVDF (piezoelectric sensing techniques), this paper attempts to study the propagation law of shock waves in brittle materials during the process of three-wavelength laser irradiation of polysilicon, and discusses the formation mechanism of thermal shock failure. The experimental results show that the vapor pressure effect and the plasma pressure effect in the process of pulsed laser irradiation lead to the splashing of high temperature and high density melt. With the decrease of the laser wavelength, the laser breakdown threshold decreases and the shock wave is weakened. Because of the pressure effect of the laser shock, the brittle fracture zone is at the edge of the irradiated area. The surface tension gradient and surface shear wave caused by the surface wave are the result of coherent coupling between optical and thermodynamics. The average propagation velocity of laser shock wave in polysilicon is 8.47 × 10^3^ m/s, and the experiment has reached the conclusion that the laser shock wave pressure peak exponentially distributes attenuation in the polysilicon.

## 1. Introduction

A PVDF (piezoelectric sensing techniques) piezoelectric sensor, which has been developed in recent years with a high response frequency (nano_second level) and above a 20 GPa pressure measurement range, is an ideal test component of laser shock waves [1]. Researchers have successively measured the attenuation law of laser shock waves in the solid target, shock wave velocity and stress wave waveform [2]. Silicon materials are excellent optical materials, usually used in the filter and substrate materials of optical systems, and they are widely applied in the microelectronics industry, optoelectronic industry and other fields. Because the band width of silicon materials is narrower than other materials (1.12 eV at 300 K), they have a large intrinsic absorption of the infrared band laser. Meanwhile, they are brittle materials with a narrow plastic region, so they are prone to be damaged in the infrared wavelength of strong light irradiation [3]. Chen et al. [4] used a laser to etch microchannels on the surface of polysilicon to increase photoelectric conversion efficiency of polysilicon solar cells. The results show that the efficiency of polysilicon solar cells and microchannels is increased by 0.23%–1.50%. The fill factor of microchannel scanning also improves polycrystalline silicon solar cells. Karnakis et al. [5] used nanosecond Nd:YAG (Neodymium-doped Yttrium Aluminium Garnet) at 355 nm high-intensity laser etching of monocrystalline silicon. The results show that an abnormally high etching depth is observed on the silicon surface when the intensity of the incident laser exceeds a certain threshold. Mainly due to a high-intensity laser explosive boiling mechanism with the secondary heating of the plasma, the laser energy changes and comes up with two different erosion mechanisms. Dobrzanski et al. [6] used the wavelength of a 1064 nm laser to treat the micro-texture of the solar polysilicon surface, which improved the solar cell trapping, effectively reducing the reflectivity and improving the efficiency of solar cells. Kumar et al. [7] studied the microstructure evolution of a silicon surface by laser etching. Therefore, it is of great practical significance to study the damage mechanism of silicon with different wavelengths.

## 2. Theoretical Analysis of Thermodynamic Effects of Polycrystalline Silicon Irradiated by Single Pulse Laser

### 2.1. Analysis of Vapor Pressure Effect

When the laser is irradiated to the surface of polysilicon, the laser is absorbed strongly. When the time waveform of output laser is Gaussian, the change of temperature rise with pulse time can be described as follows:

(1) $$T(x,t)=\frac{2AI_{0}}{k}\sqrt{DI}erfc\left(\frac{x}{2\sqrt{Dt}}\right),\;t<t_{p}.$$

When the pulse is completed, the temperature rise equation is:

(2) $$T(x,t)=\frac{2AI_{0}}{k}\left\{\sqrt{DI}erfc\left(\frac{x}{2\sqrt{Dt}}\right)-\sqrt{D(t-t_{p})I}erfc\left[\frac{x}{2\sqrt{D(t-t_{p})}}\right]\right\},\;t\geq t_{p}.$$

When the laser is continuously irradiated to polycrystalline silicon, the surface temperature rises to the evaporation temperature of the target, which will build up pressure on the target as the target material evaporates. Assuming that the $p_{\upsilon }(T_{s})$ temperature is the equilibrium vapor pressure, the expression is expressed as [8,9]:

(3) $$p_{\upsilon }(T_{s})=P_{\infty }\exp\left[\frac{L_{\upsilon }}{k_{B}T_{s}}\left(\frac{T_{s}}{T_{b}}-1\right)\right],$$

where $T_{s}$—target surface temperature, $T_{b}$—boiling point temperature, $p_{\infty }$—equilibrium vapor pressure at $T_{b}$—temperature, $L_{\upsilon }$—latent heat of vaporization, and $k_{B}$—Boltzmann constant. It is obvious that target surface vapor pressure is related to temperature, and it is a function of temperature.

### 2.2. Plasma Shock Wave Pressure Effect

Plasma generation occurs when a pulsed laser is irradiated on a target, which includes a plasma flash and a plasma blast process to produce a shock wave [10]. A plasma shock wave propagates against the incident direction in the form of ultrasound, which will have a certain pressure effect on an irradiated area of the target. According to Phipps’s pressure load analysis formula, we can get the pressure formula of the plasma shock wave on the surface of laser target area [11]:

(4) $$P=bI{\left(I\lambda \sqrt{\tau }\right)}^{n}.$$

In the formula, λ (nm) is the laser wavelength, $I_{a}{\;(\mathrm{GW}/\mathrm{cm}}^{2})$ is the laser power density, τ (ns) is the laser pulse width, and *b* is the parameter determined for the material *n* = −0.3, *b* = 5 [12].

## 3. Experimental Equipment and Methods

**Figure 1:**
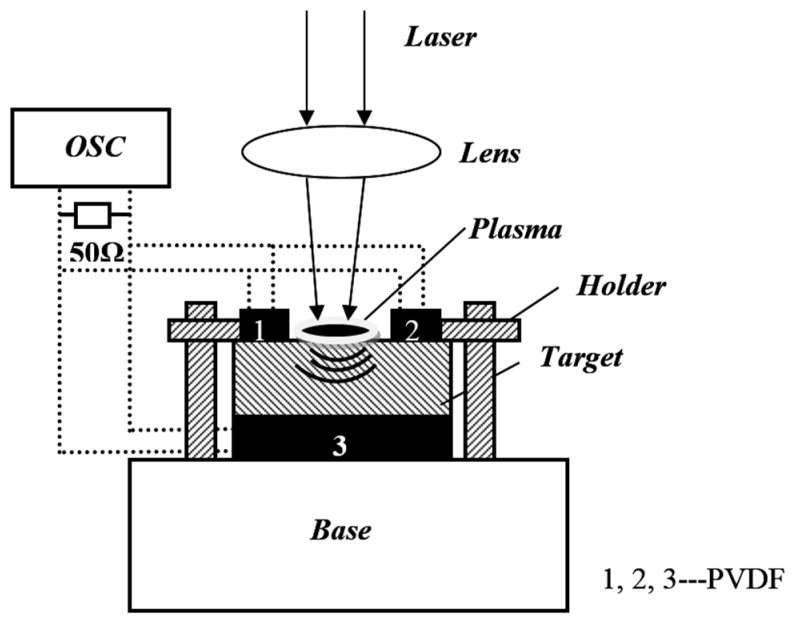
Shock wave measurement experiment device schematic.

The experiment uses the polysilicon sample of 30 mm × 30 mm × 0.25 mm and removes oil on the sample surface with acetone, cleans it with ethanol, and dries it with cold air. Taking the effect of laser thermal effects into account, according to the literature [13] on temperature and location relationship of laser ablation silicon material, when the temperature at a depth of 1.0 × 10^−6^ m is 300 K, its thermal impact on PVDF can be ignored. The target surface has no absorbing and constrained layers, but PVDF piezoelectric sensors are attached to it and perpendicular to each other, and the back surface of the target is closely fitted with PVDF through the clip. PVDF has a very thick base that can absorb the shock wave through PVDF. The Nd:YAG solid-state lasers (1064 nm, 532 nm, 355 nm), pulse width of 10 ns, laser spot diameter of 2 mm, and pulse laser energy range of 100–200 mJ are used in the experiment. The acquisition device of shock wave pressure signal is shown in Figure 1.

## 4. Experimental Results and Analysis

### 4.1. Vapor Pressure Effect on the Target Surface

**Figure 2:**
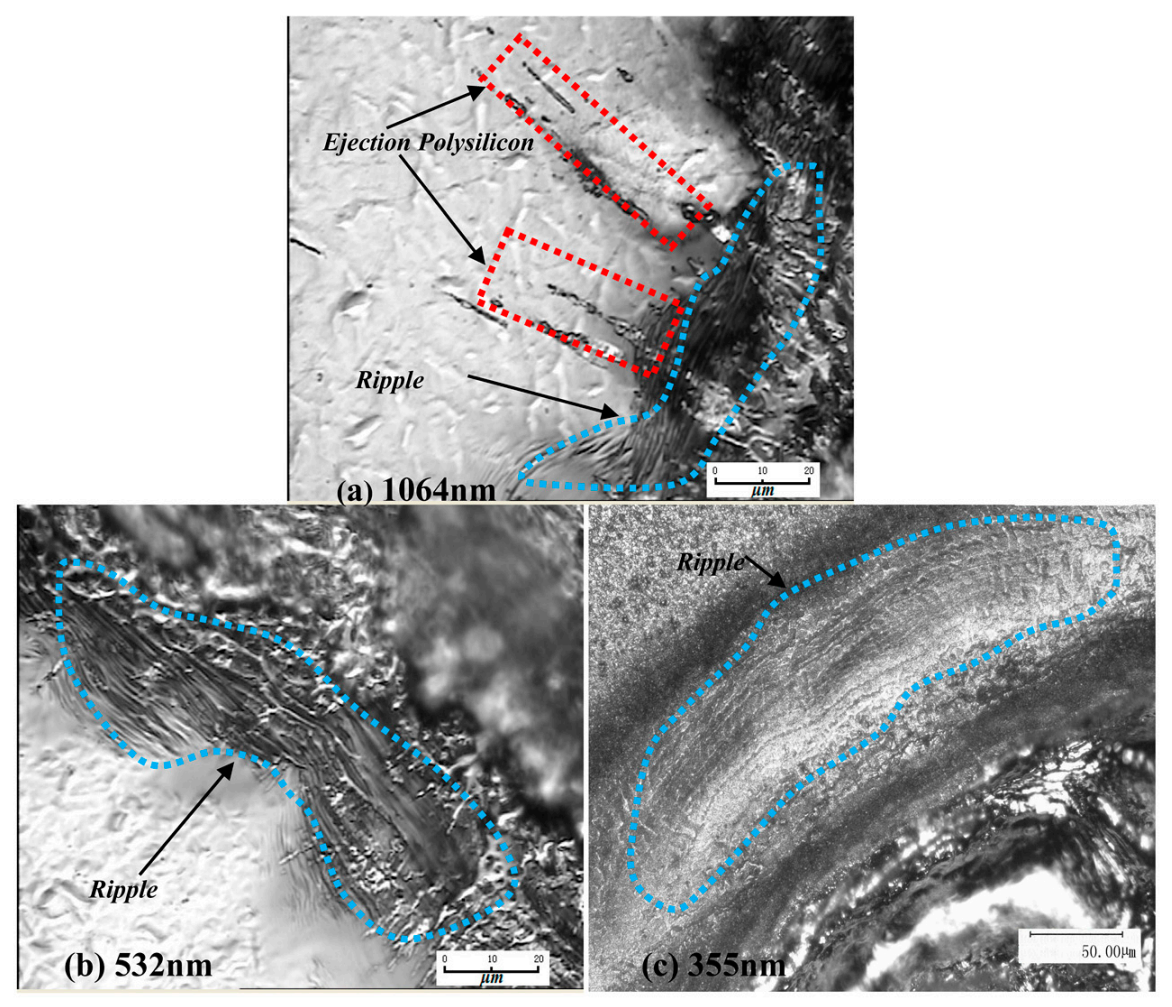
The ripples of three wavelengths after laser irradiation.

**Figure 3:**
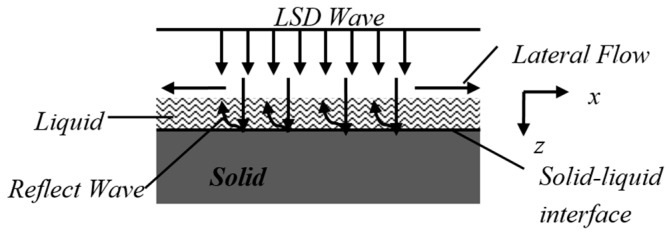
Interactions of the silicon melting layer and solids by LSD wave.

When using laser to irradiate the target, the energy absorbing particles collide with each other to transfer energy, and the temperature of the material surface will be significantly increased after being heated by laser energy [14]. As shown in Figure 2, sputtering and wave-like ripples appear outside the boundaries of the three-wavelength laser irradiated regions due to the high temperature of the material in the irradiated region and the propagation of waves to the surroundings. When the energy density of the laser exceeds the breakdown threshold of the polysilicon, a laser breakdown occurs. The vapor formed on the surface of the material forms a substance vapor with the outward splashing substance and continues to absorb the energy, thus it is partially ionized to form a high temperature and high pressure plasma. In the process of a laser pulse, the splashing phenomenon of high temperature and high density molten material shows that the melted, gasified and ionized material is discharged acutely. Due to the further enhancement of the laser intensity, the surface of the irradiated polysilicon vaporizing, the ionized breakdown of the ambient gas, or the gasified substance produces a high-speed laser detonation wave (LSD), at which time the expansion gas and the surface melted layer interact. First, high-speed air stamps molten liquid and then couples to the solid part to form the pressure, causing liquid–solid interface deformation. At this point, the strain of the media is the result of thermal mechanical coupling. The laser shock wave causes the pressure wave in the melt layer to emit back when it encounters the solid–liquid interface. As the laser energy is large, the solution layer will peel off the solid surface and splash out, as shown in Figure 3. The melting and gasification of the polysilicon in the laser irradiation region provides the conditions for the removal of the material, but the vapor pressure contributes greatly to the migration of the material.

At 300 K, the light penetration depth coefficients ($\alpha^{-1}$) of polysilicon materials with different wavelengths (1064 nm, 532 nm, 355 nm) were 0.01, 1, 1000, respectively [15]. The bandgap width *Eg* of the polysilicon is 1.12 eV. Based on the Equation: $E(eV)=h\nu =1.24\cdot \lambda^{-1}$, the laser photon energies of the three wavelengths (1064 nm, 532 nm, 355 nm) are 1.165, 2.33 and 3.49 eV. The absorption coefficients of the polycrystalline silicon material at 1064, 532 and 355 nm were 10^3^, 6.68 × 10^3^ and 10^6^ cm^−1^ [16]. Under this energy, the wavelength of 1064 nm is mainly due to hot melting damage in the photothermal mode. During the irradiation process, the molten material is greater, and the spray phenomenon occurs under the action of the vapor pressure and more obvious ripples are formed. The wavelength of 532 nm is mainly due to hot melting damage and photothermal damage (the high photon energy opens the chemical bond of the material directly and causes a photochemical reaction to cause the debris of the material to be ejected in a small or gaseous manner [17]). The main form of damage is hot melt damage and the melt is relatively less than 1064 nm, but also forms a clear ripple. The wavelength of 532 nm is mainly due to hot melting damage and photothermal damage, but the main damage is photothermal damage. The degree of ripple formation is relatively small.

When the laser irradiates the surface of the target, the laser will melt the surface layer of the target, and the pulse laser will generate the heat wave again, which causes the periodic change of the target surface [18]. Under the action of detonation wave impulse, a large radial vapor pressure will be generated, and the liquid will be pushed to the edge of the irradiated area to form a wave-like phenomenon. The high-pressure and high-speed expansion airflow stamps molten liquid and couples to the solid target part, producing the back pressure, which is perpendicular to the surface, to form the elastic wave source and produce the surface shear wave. Then, the propagation of the transverse wave causes the liquid–solid interface deformation. At the same time, the temperature gradient of the molten material forms a surface tension gradient, which aggravates the ripple phenomenon. In the modulated laser irradiation, the high temperature region of the liquid on the molten liquid surface is pulled to the low temperature region; it is the result of optical and mechanical coherent coupling. The thermal wave model theory can be used to analyze it, aiming at the phenomenon of corrugation on the silicon surface. Under the experimental conditions, the laser pulse width of wavelengths 1064, 532 and 355 nm is 10 ns; thus, the thermal wave frequency can be obtained:f≈1/2τ=50 MHz. Assuming that the stripe spacing d is wavelength λ of the thermal wave, the wave velocity can be obtained by the following Equation (5) [18]:

(5) $$v^{2}=\frac{g}{m}\tanh mh+\frac{T_{m}}{\rho },$$

where in m=2π/d, *T* is the surface tension, *g* is the gravitational acceleration, and ρ is density, and the density of the silicon material is 2.3 g/cm^3^. Since h≥d/2, *d* is small, the above Equation (6) is simplified as:

(6) $$v^{2}=2\pi \frac{T}{\rho d}.$$

Putting d=2 μm, ν=500 m/s into Equation (5), the surface tension can then be obtained as: T=183 N/m.

**Figure 4:**
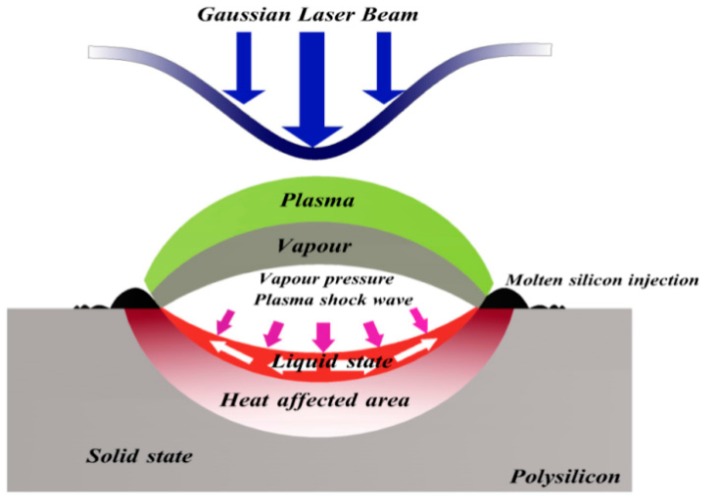
Schematic diagram of irradiation upon the polysilicon of the pulse laser.

According to the experiment phenomenon, we summarize the interaction process between a high energy pulse laser and polysilicon material, as shown in Figure 4. When distributed laser of Gaussian irradiates polysilicon, the energy of the light spot center is the largest, so the center of the irradiation area is the area of peak parameter. As shown in the Figure 4 laser irradiation area, due to the effect of a high energy laser, the silicon material changes from solid to liquid on the surface of the target, and moves toward the light spot edge under the action of plasma pressure and steam pressure. Then, the melt recrystallizes again and becomes solid on the edge of the light spot. On top of the liquid state material is the steam, not fully ionized. On top of the steam is the plasma layer. Because the area is a completely ionized material, the plasma layer will produce high pressure to compress the steam layer, and accelerate the ionization of the molecular state material. Under the liquid state is the heat affected zone and under it is the solid area that is not affected by the laser.

### 4.2. Effect of Plasma Shock Wave Pressure upon the Surface of the Target

#### 4.2.1. Shock Damage Location of the Target Material Surface under the Laser Thermal 

**Figure 5:**
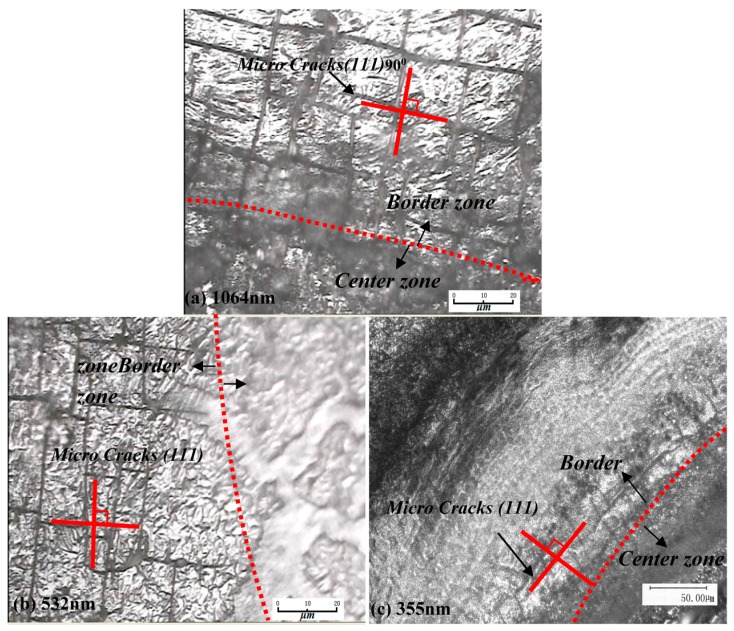
Cleavage failure under laser shock waves.

As in the above picture, the irradiation damage phenomenon under the power density 6.3 GW/cm^2^ of three wavelength lasers are shown. Because of the polysilicon materials on the (111) surface, bonding strength is the lowest between atomic bonds, and a cross type of crack damage area can be seen from Figure 5. Under the effect of the laser shock wave, the priority, and destroyed front are mutually vertical. The angle is 900, and this is due to (111) obeying the C2 symmetry, which is called brittle intergranular fracture. As shown in Figure 5, however, the cleavage damaged area does not give priority to light spot center, and the maximum degree position is on the verge of the irradiated area. The main reason is the effect of laser shock wave pressure, and we will analyze the longitudinal stress signals during the process of shock waves and lateral strain in the following part.

#### 4.2.2. The Dynamic Curve of Laser Shock Wave toward the Target 

PVDF piezoelectric film, with measuring range from 0 to 20 Gpa, with a nanosecond as its frequency response, and dynamic calibration being simple and fast, is an ideal sensor of super-high pressure measurement [19]. At time *t*, PVDF measured voltage signal *V*(*t*) and the shock pressure on the surface of PVDF thin film *P*(*t*) within the scope of $P\in [0,3\times 10^{8}\mathrm{Pa}]$ will meet the relation [20]:

(7) $$P(t)=\frac{K}{A}\int_{0}^{t}\frac{V(t)}{R}dt.$$

For dynamic calibration coefficient, *K*, its value is 6.6 × 10^8^ Pa·cm^2^/μ*_c_*, *A* is the effective area of PVDF, R is parallel resistance with PVDF, and the resistance is 50. By Equation (7), a voltage signal detected by an oscilloscope can be transformed to the laser-induced shock wave pressure signal, which, after transformation, turns out to be the actual shock wave relative pressure values.

**Figure 6:**
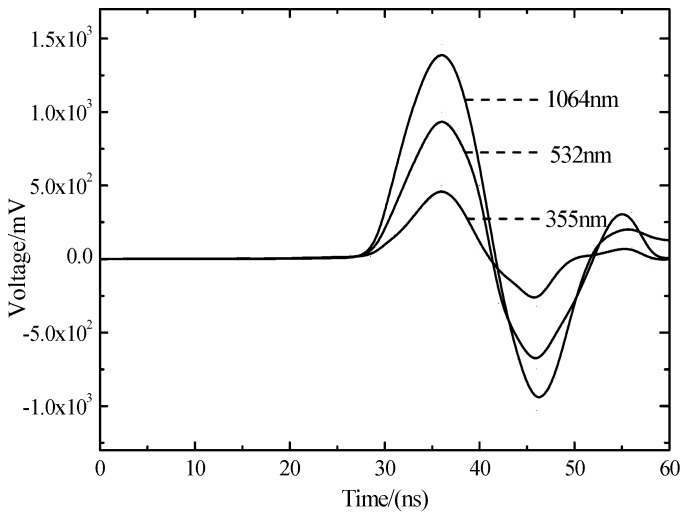
The laser shock wave piezoelectric signal of three different wavelengths.

When the pulse laser irradiates target material and produces shock waves on the workplace surface and produces reflection and transmission, the transmission wave becomes the form of stress wave in the target material. The stress wave transmits to the interior of the target material and bounces back and forth. When laser shock waves transmit to the back of the target material, it will produce a voltage pulse on PVDF piezoelectric film, and the oscilloscope will record the voltage signal. When laser power density is 6.3 GW/cm^2^, the first waveform cycle of the three wavelength laser shock is shown in Figure 6. With the decrease of wavelength, oscilloscope detected shock wave piezoelectric signal amplitude decreases.

**Figure 7:**
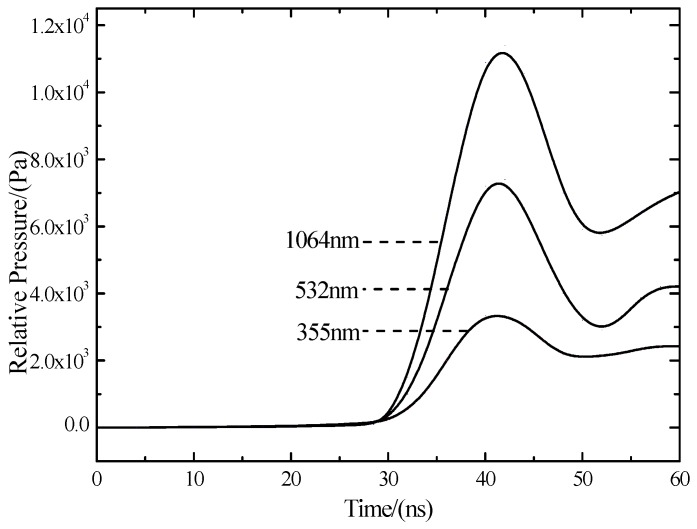
Three wavelengths of relative pressure curves.

The curve in Figure 6 and time integrates and becomes relative pressure curve Figure 7. From Figure 7 under the same power density, relative pressure of the wavelength of 1064 nm is the greatest. With the decrease of the laser wavelength, shock wave pressure of laser ablation becomes smaller. The short wavelength laser has the following characteristics: the greater photon energy, the greater the absorption coefficient, the shallower the penetration depth, the stronger the interaction mechanism of laser photons-silicon becomes, making a short wavelength laser and polycrystalline silicon couple more fully. The wavelength of 1064 nm has a high breakdown threshold. When the laser energy density is a constant value, as the wavelength from 1064 nm decreases to 355 nm, the photons and silicon coupling degree increases to strengthen the formation of shock waves.

**Figure 8:**
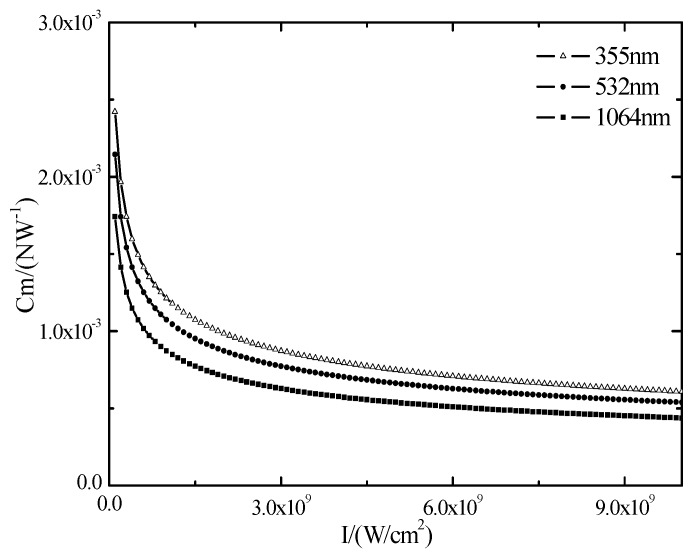
The relationship between laser parameters and coupling coefficient of the polysilicon.

However, the decrease of wavelength results in the decrease of critical breakdown power threshold and decreases laser-induced shock wave pressure, which limits the formation of shock waves [21]. According to the empirical formula presented by Phipps [11]: $C_{m}=b{\left(I\lambda \sqrt{\tau }\right)}^{n}$ *b* = 5, *n* = 0.3), the related parameters of silicon are put in and are shown in Figure 8.

As can be seen from Figure 8, laser wavelength has a great influence on the impulse coupling coefficient. When the laser power density and laser pulse width is constant, with the increase of the laser wavelength, the impulse coupling coefficient decreases. The wavelength of a 355 nm laser and polycrystalline silicon coupling degree is the largest and has highest efficiency. However, with the increase of laser power density, the impulse coupling coefficient is declining to an equilibrium state. The main causes of this phenomenon are due to an excessive steam layer and plasma spray produced by a high energy laser. Subsequently, the laser cannot penetrate and produces plasma shielding. The explosion of steam and plasma produced by laser irradiation on polysilicon make the target material surface produce recoil pressure and impulse. High energy nanosecond pulse laser works on the surface of the target material and the pressure pulse duration is very short, and its mechanical effect can be characterized with impulse, and impulse is one of the important parameters of shock wave mechanics’ effect, which can be expressed as [20]:

(8) F=∫P(t)dt.

**Figure 9:**
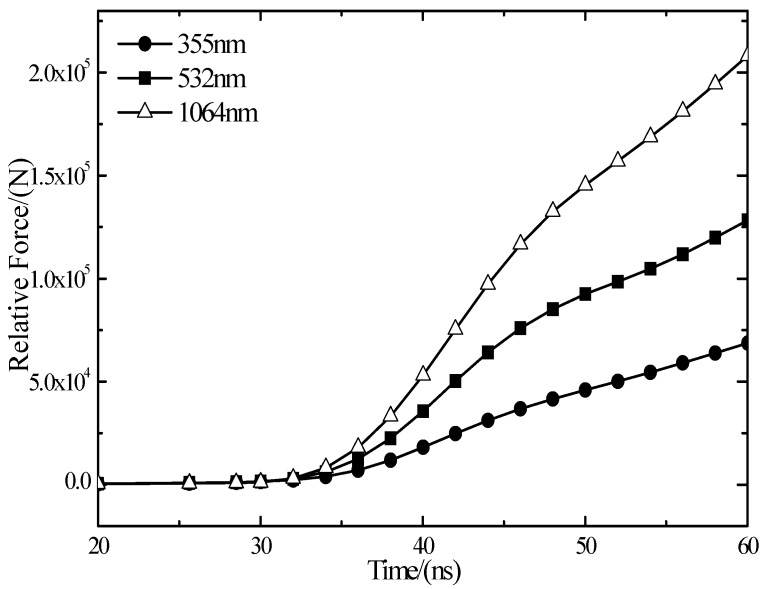
The relationship between impulse and time by different wavelengths.

Figure 9 shows the relations of ablation impulse of three wavelength single pulse lasers and times when the laser power density is 6.3 GW/cm^2^. It is through integration of shock wave pressure of different wavelengths and time curves. As can be seen from Figure 9, with the increase of pulse time, the impulse is to linearly increase. With the increase of wavelength, ablation impulse strength increases. Due to the longer wavelengths, ablation impulse is greater than the short wavelength, leading to the most serious fracture degree of the surroundings of the 1064 nm wavelength irradiation area.

#### 4.2.3. Dynamic Strain of Laser Irradiation Light Spot of Target Edges 

Using PVDF piezoelectric film sensors to collect the dynamic wave signal of the laser irradiation polysilicon plane, the strain response of PVDF piezoelectric film is transverse strain, the longitudinal strain’s effect and algebra and Equation (9):

(9) $$Q=(d_{31}\varepsilon_{1}+d_{32}\varepsilon_{2})E_{\mathrm{PVDF}}S.$$

Among them, $\varepsilon_{1}$ and $\varepsilon_{2}$ are the two perpendicular directions, and strain can be calculated through the detection of PVDF piezoelectric film on the charge transfer. The time transfer between charge and voltage signal *V*(*t*) on PVDF piezoelectric film satisfies:

(10) $$Q(t)=\int_{0}^{t}\frac{V(t)}{R}dt.$$

The output voltage signal *V*(*t*) produced by the oscilloscope in Equations (8) and (9), the $\varepsilon_{1}$ − *t* curve and the $\varepsilon_{2}$ − *t* curve can be obtained.

**Figure 10:**
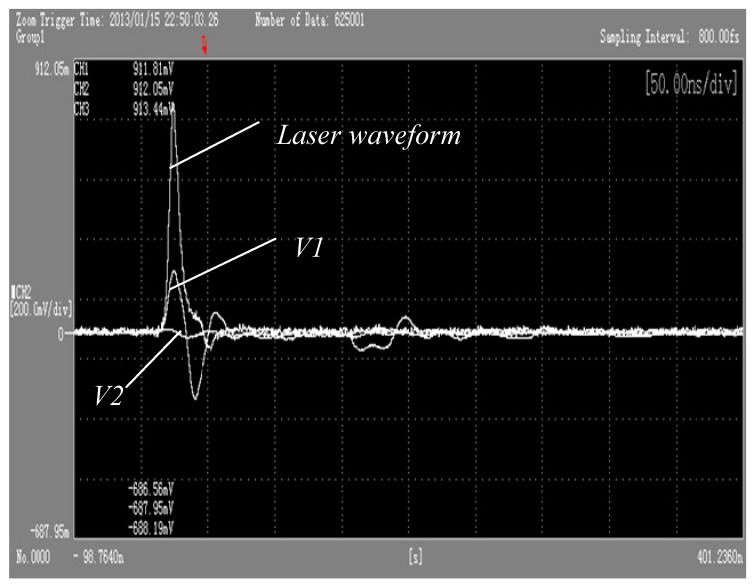
Piezoelectric signal curve when the wavelength is 1064 nm and laser power density is 6.3 GW/cm^2^ on the target surface.

**Figure 11:**
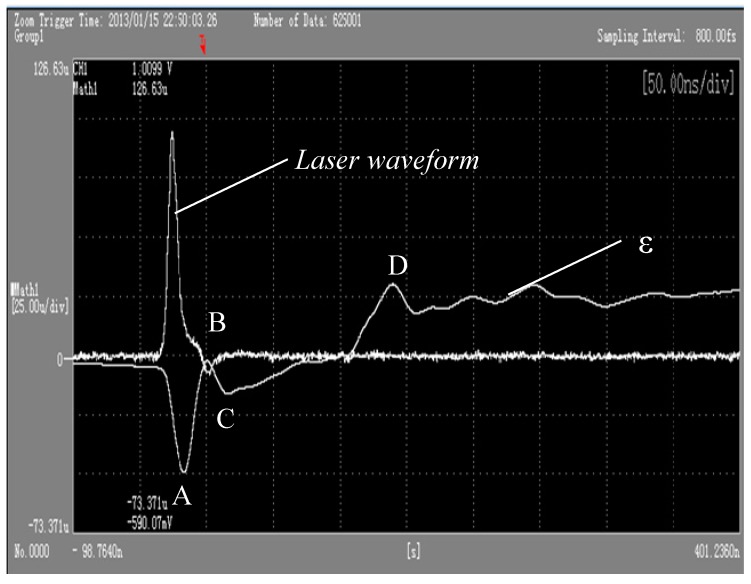
A strain curve of the target surface when the wavelength is 1064 nm and laser power density is 6.3 GW/cm^2^.

Figure 10 and Figure 11, respectively, show the oscilloscope outputs’ corresponding surface target material piezoelectric signal curve and strain curve when the wavelength is 1064 nm, and laser power density is 6.3 GW/cm^2^. In Figure 10, through the calculation by Equations (8) and (9) of the voltage signal, the laser irradiation of the polysilicon dynamic strain response curve can be produced. It can be found from Figure 10, under the action of the pulse laser, that material along the laser speckle phase radial strain (V1) and vertical direction strain (V2) change with the same trend. Laser waveform half-width is about 10 ns, consistent with the laser pulse width. In a single pulse laser action time, PVDF patch sensors detect firstly that surrounding material of the irradiated area produces compressive strain because of compression, and then compressive strain decreases in tensile strain. With the passage of time, the state of dynamic strain curve around the irradiation area is leveled off.

In Figure 11, it takes the curve ε along the radial direction of laser speckle as an example to interpret the above phenomenon. Laser irradiation in an unconstrained mode irradiates silicon and produces shock wave pressure, and the material surface of the irradiation area produces thermal expansion due to the strong absorption of laser energy, causing surface thermal stress waves. Material particles in the irradiation area under the action of the plane wave expand, making the material in the irradiation area be in the compression state. Therefore, PVDF sensors detect that the compressive strain and compressive strain increase with the increase of laser shock wave pressure. When the laser shock wave is up to the peak pressure, compressive strain is corresponding to a point on the curve ε. Then, within the time pulse width, as the time continues to the end, the laser temperature drops, and the laser shock wave pressure decreases, entering the stage of pressure unloading. Then, compressed material begins to rebound, leading to the compressive strain decrease. When the rarefaction wave spreads to the center of the impact zone, compressive strain decreases to point B. However, when the longitudinal stress wave returns to the surface of the target from the back of the target material, anti-media of high impedance (poly) spreads to anti-media of low impedance (air), which leads to the stress wave of the laser transforming into an elastic rarefaction wave and makes the material on the surface compress to point C under the action of tension. In addition, point D in the figure is the result of impacts of the following longitudinal wave. Finally, with the attenuation of the shock wave, strain tends to achieve a balance.

**Figure 12:**
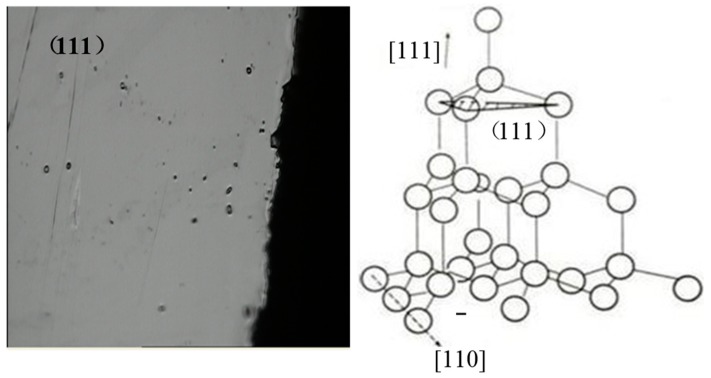
Transgranular cleavage failure section and structure.

It can be analyzed by combining with analysis of how PVDF detects the curve of stress waves on the surface as follows: under the irradiation of a high energy laser, polysilicon is induced to produce a plasma shock wave by laser ablation, and the shock wave produces great pressure on the target. As a result, the material at the edge of the radiating area transfers from compression to tension in a short time, in that it is firstly influenced by compressive stress of the thermal expansion wave. Furthermore, under the irradiation a Gaussian beam of light, the maximum tensile stress appears at the edge of the area, forming the effect of steam pressure and that of plasma shock wave, owing to the ablation under the high energy laser. At the same time, the center of the laser irradiation area forms softening effects after receiving the high energy laser energy, and then it has the tendency to move forward under the action of shock waves, making the material around the spot center follow its displacement. However, polysilicon belongs to brittle materials that have a narrow plastic zone, and the transgranular cleavage is broken due to the sudden appearance of tensile stress. In addition, a softening effect to a certain extent of the material in the center of the laser irradiation area leads to a slight increase in the width of the plastic zone, and makes the material which is at the edge of the area appear to have the narrowest plastic zone. Therefore, when the material that is at the center of the area has a tendency for displacement, the degree of damage is higher. In Figure 5a (1064 nm), there appear a large number of micro cracks at the edge of the irradiation area, and the micro cracks in Figure 5c (355 nm) have less damage compared with the other two wavelengths. In conclusion, the reason why transgranular cleavage is broken is that the shock wave effect, which is induced by the laser, pressure effect and thermal coupling effect, act jointly. Figure 12 shows the surface topography of cleavage damage fracture (111) and crystal structure. It can be seen from the diagram that the fracture, which has the weakest binding force of atomic link in the silicon material, appears to be smooth and flat and will be damaged firstly under the action of the laser shock wave. In the diagram, the face of [111] is perpendicular to the (111) plane.

According to the analysis formula of pressure overload put forward by Phipps [11], the semi-empirical formula of pressure can be concluded, which is about the pressure produced by the laser plasma shock wave to the target surface in the laser irradiation area:

(11) $$P=bI{\left(I\lambda \sqrt{\tau }\right)}^{n}.$$

**Figure 13:**
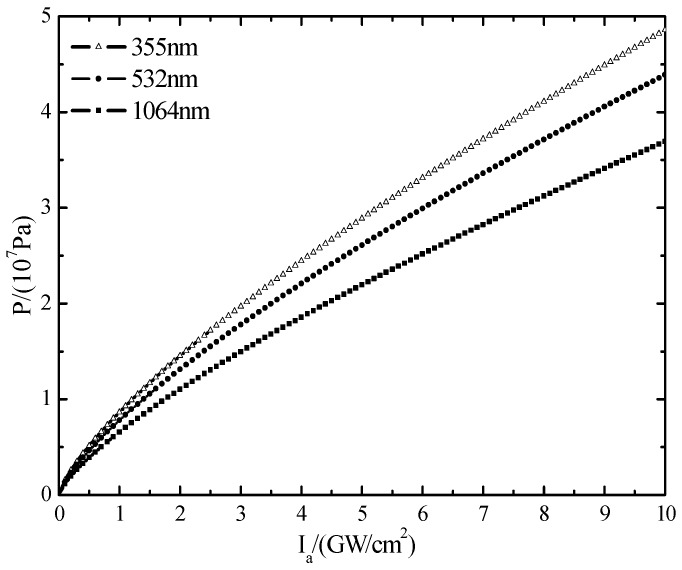
A diagram which shows the relationship of pressure laser wavelength and power density.

In Equation (11), λ (nm) is laser wavelength, $I_{a}{\;(\mathrm{GW}/\mathrm{cm}}^{2})$ is the laser power density, τ (ns) is the laser pulse duration, and *b* are the parameters of the silicon material determined by the material—*b* = 5. In addition, Figure 13 can illustrate Equation (11).

The relationship between laser wavelength and pressure of target surface in the laser irradiation area can be fully expressed in Figure 13. In theory, with the increase of laser power density, the pressure is enhanced, and with the decrease of the wavelength, the pressure on the surface becomes greater. However, the energy of the laser photon that belongs to short wavelengths is great and a full consideration of the coupling between the short wavelength and material has been achieved. As a result, the breakdown threshold values of polysilicon are reduced, and when the laser energy is greater than the breakdown threshold, the excess energy will ionize the material in the irradiation area that is in the gaseous, atomic state quickly, so as to reduce the possibility of shock wave formation. Meanwhile, the experimental phenomena is consistent with the research results about regularities of distribution of silicon’s laser plasma pressure, which is carried out by Bao et al. [22]. The experimental phenomena shows that the spot center has the biggest pressure, and it will have a tendency of displacement due to the pressure, driving the material that is at the edge of the area. However, because of the brittle material’s compressive strength being greater than tensile ability, the material at the edge of area will crack due to the great tensile stress.

### 4.3. The Attenuation Rule of Laser Shock Wave in Polysilicon

**Figure 14:**
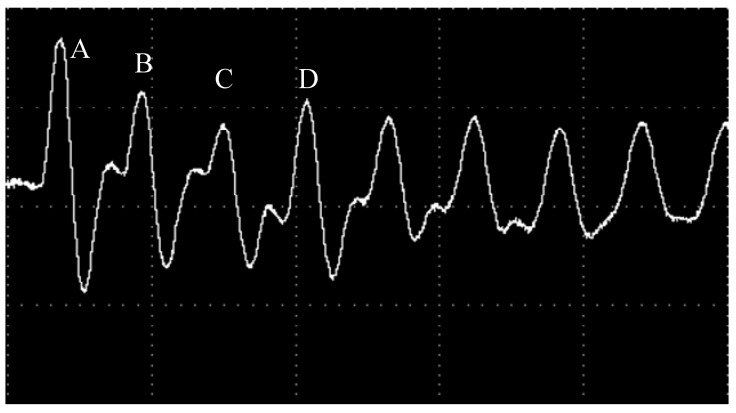
The piezoelectric waveform of shock waves that are formed by the laser’s 1064 nm wavelength inducing silicon’s target surface.

The piezoelectric waveform, whose laser power is 6.3 GW/cm^2^ and the wavelength is 1064 nm, has the biggest amplitude, and is similar to the other waveforms. Then, we take the 1064 nm wavelength as an example to analyze its periodicity. As shown in Figure 14, it can be found that the biggest peak is formed by a shock wave that is induced by a laser and the reflection of following wave. Meanwhile, the average speed of shock waves spreading in polysilicon can be calculated according to their periodicity. As shown in Figure 11, the duration between the two neighboring pulses of voltage waveform is *t*, which means the time interval between the two adjacent laser shock waves reaching the target material on the surface. It can be read from Figure 11 that *t_AB_* = 55 ns, *t_BC_* = 60 ns, and *t_CD_* = 61 ns. We can take the average: *t_ave_* = 59 ns, and the thickness of the sample can be illustrated as follows: *d* = 0.25 mm. Therefore, according to the formula: V=2d/t, the average speed of shock waves spreading in the sample of polysilicon can be concluded as *v* = 8.47 × 103 m/s. Because shock waves induced by laser transmit back and forth in the polycrystalline silicon sheet, the unconstrained and absorbed layer of a polysilicon target’s front surface is the free surface. However, the acoustic impedance of polysilicon is six orders of magnitude larger than the acoustic impedance of air. Thus, the free surface can be regarded as the free end of reflection, and the size of wave pressure, which is formed after reflection, remains the same. Then, as the area of a polysilicon sheet’s rear surface where the laser will function is pasted up with a PVDF membrane, part of the shock wave will reach the base after a transmission through PVD membrane. In addition, the base will absorb the shock wave that transmits through the PVDF. Compared with the thickness of polysilicon target material, the thickness of base can be ignored and the shock wave requires more time to reflect in the base than attenuate in the target material. Therefore, the transmission wave will not affect the accuracy of experimental results. However, the loss part of the shock wave that transmits into the PVDF piezoelectric membrane needs a certain amount of compensation while measuring shock wave pressure. Dividing the actual peak pressure values of shock waves, which are formed by reflecting on the polysilicon material surface many times, by the formula: $F^{n}$, the peak voltage without transmission loss can be concluded [23]:

(12) $$F^{n}={\left(\frac{Z_{si}-Z_{\mathrm{PVDF}}}{Z_{si}+Z_{\mathrm{PVDF}}}\right)}^{n}.$$

In Equation (12): $Z_{si}=1.239\times 10^{6}\;g\cdot {\mathrm{cm}}^{-2}\cdot s^{-1}$, $Z_{\mathrm{PVDF}}=0.25\times 10^{6}\;g\cdot {\mathrm{cm}}^{-2}\cdot s^{-1}$, F=0.96.

As mentioned above, the shock waves induced by the laser reflect continuously on the anterior and posterior surface of the polycrystalline silicon sheet. Therefore, when the shock wave reflects on the anterior and posterior surface of polycrystalline silicon, the distance can be used to draw an attenuation rule of the peak pressure of shock waves. In addition, the peak pressure of shock waves is illustrated in Table 1.

**Figure 15:**
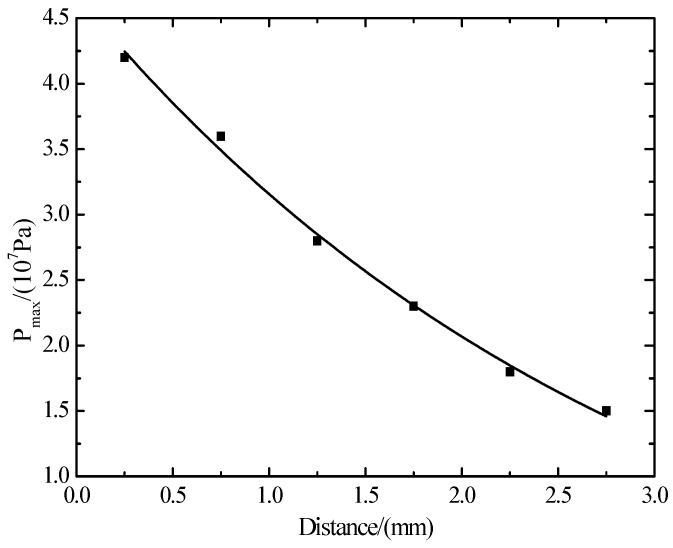
The attenuation rule of shock wave pressure in polysilicon.

The attenuation rule of laser shock wave’s peak values in polysilicon is concluded by the method of exponential decay fitting (Figure 15):

(13) $$P_{\max}=5.37\exp\left(-\frac{x}{3}\right)-0.7.$$

In the formula,*P*_max_ is the shock wave’s peak pressure and *x* is the shock wave’s spreading distance.

According to Equation (12), we can conclude that the maximum peak value is 4.67 × 10^7^ Pa.

## 5. Conclusions

Based on the detection techniques of the PVDF piezoelectric membrane, this paper studies laser thermal shock’s devastating phenomenon in the process of laser irradiating polysilicon material and analyzes the function of wavelength effect in the process of shock waves, which is induced by a laser on the polysilicon material damage. Meanwhile, this paper also uses the test results of PVDF piezoelectric films to analyze the failure mechanism during the process of laser irradiating polysilicon and studies the propagation’s law of laser shock wave rules that is in the brittle materials. Thus, the main results are as follows:

1. In the process of pulsed laser action, the combined action of steam pressure and plasma contributes to splash phenomenon of melt, which has a high temperature. In addition, the melting and gasification of polysilicon in the area of laser irradiation provide conditions for the removal of material. Furthermore, the steam pressure and plasma pressure have made great contributions to the removal of material. In addition, the decrease of the laser wavelength enables stronger coupling between laser and target, reducing the breakdown threshold value and weakening laser shock wave strength.
2. When the speedily expanding airflow of high pressure stamps melts and couples to the solid part of the target material, recoil pressure will be produced that is perpendicular to the surface, and elastic waves will be composed to form transverse waves on the surface. Furthermore, the spread of transverse waves will cause the deformation of the liquid–solid interface, which is similar to the formation of water waves. At the same time, the tension gradient that is on the surface of the target material causes the phenomenon of moiré, and the higher the temperature of the molten material is, the smaller the surface tension will be; in addition, the lower the temperature is, the greater the surface tension will be. Under the modulated laser irradiation, the liquid on the high temperature zone is pulled into the low temperature zone, which is a result of the coherence and coupling between optics and mechanics.
3. Brittle material has a poorer ability to resist shear stress, so it is easy to find the phenomenon of cleavage destruction under the effect of laser shock waves, and the destroyed area is just the edge area of the laser irradiation.
4. The average spreading speed of shock waves in the sample of polysilicon is v = 8.47 × 10^3^ m/s, and the attenuation tendency of the laser shock wave’s pressure peak in polysilicon is in the form of exponential distribution.

## Figures and Tables

**Table 1:** The peak voltage which is formed after laser shock waves’ reflection on 0.25 mm wafers.

Thickness/mm	0.25	0.75	1.25	1.75	2.25	2.75
Relative peak voltage/10^7^ Pa	4.2	3.5	2.7	2.2	1.7	1.4
Peak voltage withouttransmission loss/10^7^ Pa	4.2	3.6	2.8	2.3	1.8	1.5
